# Determining the potential distribution of *Oryctes monoceros* and *Oryctes rhinoceros* by combining machine-learning with high-dimensional multidisciplinary environmental variables

**DOI:** 10.1038/s41598-022-21367-1

**Published:** 2022-10-19

**Authors:** Owusu Fordjour Aidoo, Fangyu Ding, Tian Ma, Dong Jiang, Di Wang, Mengmeng Hao, Elizabeth Tettey, Sebastian Andoh-Mensah, Kodwo Dadzie Ninsin, Christian Borgemeister

**Affiliations:** 1 Department of Biological, Physical and Mathematical Sciences, School of Natural and Environmental Sciences, University of Environment and Sustainable Development, Somanya, Ghana; 2 State Key Laboratory of Resources and Environmental Information Systems, Institute of Geographic Sciences and Natural Resources Research, Chinese Academy of Sciences, Beijing, 100101 China; 3 College of Resource and Environment, University of Chinese Academy of Sciences, Beijing, 100049 China; 4 Council for Scientific and Industrial Research (CSIR), Oil Palm Research Institute, Coconut Research Programme, P. O. Box 245, Sekondi, Ghana; 5 Centre for Development Research (ZEF), University of Bonn, Genscherallee 3, 53113 Bonn, Germany

**Keywords:** Ecology, Plant sciences, Zoology, Climate sciences, Ecology, Environmental sciences, Energy science and technology, Mathematics and computing

## Abstract

The African coconut beetle *Oryctes monoceros* and Asiatic rhinoceros beetle *O. rhinoceros* have been associated with economic losses to plantations worldwide. Despite the amount of effort put in determining the potential geographic extent of these pests, their environmental suitability maps have not yet been well established. Using MaxEnt model, the potential distribution of the pests has been defined on a global scale. The results show that large areas of the globe, important for production of palms, are suitable for and potentially susceptible to these pests. The main determinants for *O. monoceros* distribution were; temperature annual range, followed by land cover, and precipitation seasonality. The major determinants for *O. rhinoceros* were; temperature annual range, followed by precipitation of wettest month, and elevation. The area under the curve values of 0.976 and 0.975, and True skill statistic values of 0.90 and 0.88, were obtained for *O. monoceros* and *O. rhinoceros*, respectively. The global simulated areas for *O. rhinoceros* (1279.00 × 10^4^ km^2^) were more than that of *O. monoceros* (610.72 × 10^4^ km^2^). Our findings inform decision-making and the development of quarantine measures against the two most important pests of palms.

## Introduction

The African coconut beetle *Oryctes monoceros* (Olivier) and the Asiatic rhinoceros beetle *O. rhinoceros* (L.) are sap-sucking coleopteran pests of palms. The rhinoceros beetles are, economically, the greatest threat to the palm industry^1^. The two species have similar biology and ecology^2^. Adult beetles bore into the apical section of the palm through the basal parts of the leaves and enter the heart of the unfolded leaves, inflicting physical damage at the growing point of the infested palm. The damage may lead to the subsequent death of the palm^3^. In tropical Africa, *O. monoceros* can cause up to 40% damage to coconut^2^. The estimated losses of *O. rhinoceros* to coconuts in India and Indonesia are approximately 10%, while losses to oil palm have been estimated to be as high as 25% in Malaysia and the South Pacific regions^1,4–6^.

*Oryctes monoceros* and *O. rhinoceros* attack over 30 palm species, among which the most economically important ones are oil palm, coconut, and date palms^7^. Apart from palms, the rhinoceros beetles also attack sugarcane^8^. Management strategies for the two pests include chemical pesticides, old fishing nets for trapping adults, removal of adults with the metal hook, and destruction of breeding sites which include dead logs, cow dung, and organic manure. Biological control using the entomopathogenic fungus *Metarhizium anisopliae* has been proved useful in reducing the population of *O. rhinoceros* in the Philippines and Indonesia^9,10^. The entomopathogenic *Oryctes rhinoceros* nudivirus (previously known as *Baculovirus oryctes*) has provided control of invasive populations of *O. rhinoceros* in the Pacific islands^6^ and has also been tested in Tanzania with less success^11^.

*Oryctes monoceros* undergoes a complete metamorphosis. Its breeding sites are found in rotten logs, compost, and decaying vegetation. Adults lay eggs in these organic materials and subsequently hatch into 1st instars, the latter taking about 10–13 days. The 1st larval stage may take 9–20 days to develop into the 2nd instar^12^, and about 34–54 days thereafter to move into the 3rd instar. The final stage where pupae develop into adults requires about 20–30 days, depending on food availability and prevailing environmental conditions. *O. monocero*s may live for up to six months^12,13^. Similarly, *O. rhinoceros*, undergoes a complete metamorphosis. Its eggs hatch in 8–12 days, and after that, the larvae spend their whole larval stage inside the breeding medium. The larva requires 80–200 days to develop: the first instar takes 10–21 days, the second instar 12–21 days, and the third instar 60–125 days. The beetle then goes through an 8–13-day prepupal stage before pupating in a pupal chamber built out of the feeding substrate. Pupae last for 17–30 days, after which they emerge as adults that can live for up to 6 months or longer^14–16^.

*Oryctes monoceros* is distributed throughout the tropical regions of Africa and has been recently reported in Yemen^17^. In contrast, *O. rhinoceros* is indigenous only to South and Southeast Asia^6^ which may be due to lack of a contiguous land mass. Its major pathways of transmission have been the transportation of host materials by humans, floating logs carried with ocean currents, and the shipment of wartime equipment^18^. For instance, the spread of *O. rhinoceros* has been associated with the transport of the pest on commercial soil products^19^. At various possible entry points in the United States of America, live adults of *O. rhinoceros* have been intercepted five times, originating once from China, Malaysia, Sri Lanka, and twice from Indonesia^7^, highlighting the potential for dispersion of the beetle outside its native range.

Advancements in geographic information systems (GIS) and remote sensing technology, as well as quick advances in relative statistical modeling and analysis, have offered new indicators for biogeographical studies on pests^20^. Ecological niche models are commonly employed in forecasts of climate change to quantify its potential impacts on regional ecology and biogeography^21^. The models are a group of approaches that combine species occurrence records with environmental data to create a correlative model of the environmental variables that can meet a species’ ecological requirements and predict its potential habitat^22^. The models have been used to obtain the following outputs: (a) to assess the relative suitability of habitats known to be occupied by the species, (b) to evaluate the relative suitability of habitats in geographic areas not known to be occupied by the species, (c) to predict changes in the suitability of habitats over time given a specific scenario for environmental change, and (d) to estimate the species occurrence^23,24^. Bioclimatic Prediction and Modeling System (BIOCLIM), Genetic Algorithm for Rule-set Prediction (GARP), CLIMEX model, Random Forest (RF), Booted Regression Trees (BRT) and the maximum entropy (MaxEnt) are applied as the main ecological niche models. The latter is a machine learning algorithm that employs the theory of maximum entropy^25–27^. This model relies on known occurrence records of a species and corresponding environmental variables to analyze and predict the geographical distribution of the species when the entropy reaches the highest point under limited conditions^27^. There is a risk of over-fitting and bias in the present locations for MaxEnt modeling, and this can limit the model's performance^28,29^. However, the model has several advantages including the use of presence only records because species absence records are rarely available or reliable, the model results are continuous, which allows classification of levels of suitability in different areas, and the model uses a generative approach by incorporating environmental data from the study area thereby avoiding the need for absence data^25,27,30^. MaxEnt has a high simulation accuracy, and has been widely used in the prediction of favorable locations for insects such as *Dalbulus maidis* (DeLong) (Hemiptera: Cicadellidae)^31^, (*Coleoptera: Cerambycidae*)^32,33^, *Lycorma delicatula* White (Hemiptera: Fulgoridae)^34^, *Daktulosphaira vitifoliae* Fitch (Homoptera: Phylloxeridae)^35^, and *Planococcus ficus* Signoret (Hemiptera: Pseudococcidae)^36^.

Climate change and global warming will have a significant impact on species distribution and abundance, as well as the extent of pest losses, affecting crop output and food security^37^. Research has shown that increased temperatures, rising CO_2_ levels in the atmosphere, and changing precipitation patterns all have a substantial impact on agricultural production and agricultural insect pests^38^. Given that climate change will lead to a rise in pest outbreaks and changes in pest behavior and risk of invasion^37,39^, it is essential to understand how climate change influences the geographical distribution of agricultural pests such as *O. monoceros and O. rhinoceros*, which is a prerequisite for developing ecological friendly integrated pest management (IPM) strategies^40^. Previous studies on *O. monoceros* and *O. rhinoceros* have mainly focused on management strategies, biological characteristics, and environmental factors that influence the population dynamics and abundance^10,41^. However, risk maps for surveillance and monitoring are quite insufficient at this moment^7^. Therefore, in this study, for the first time, we have used the MaxEnt model and ArcGIS software to quantify and map the geographical distribution of *O. monoceros* and *O. rhinoceros*.

## Methods

**Figure 1:**
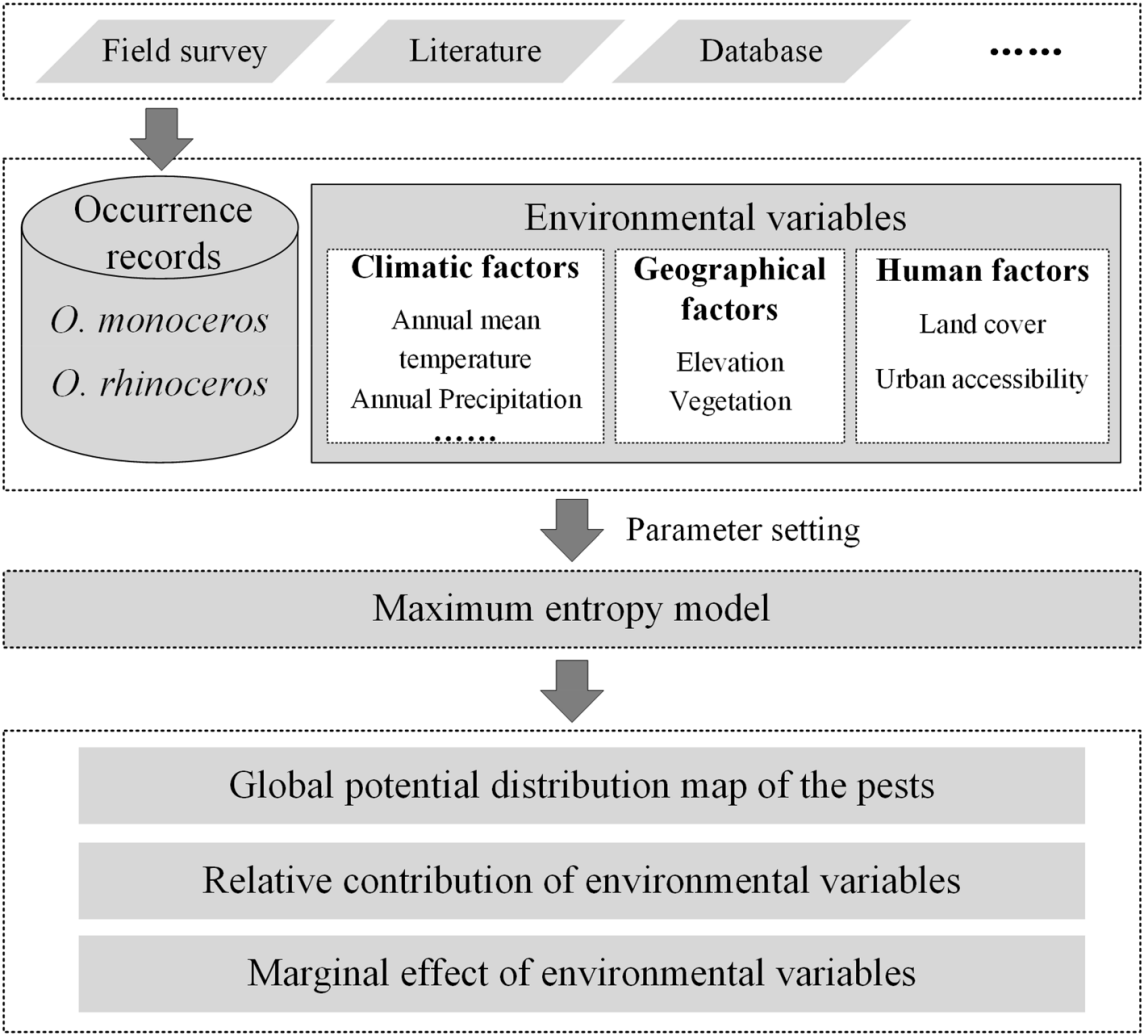
Technical flow chart of study.

We divided the analysis process into four steps: (1) acquisition of occurrence records; (2) preparation of environment variables; (3) MaxEnt modeling; and (4) production of the potential distribution maps for *O*. *monoceros* and *O*. *rhinoceros*. The technical flow chart of our study is depicted in Fig. 1.

### Occurrence records of *O. monoceros* and *O. rhinoceros*

**Figure 2:**
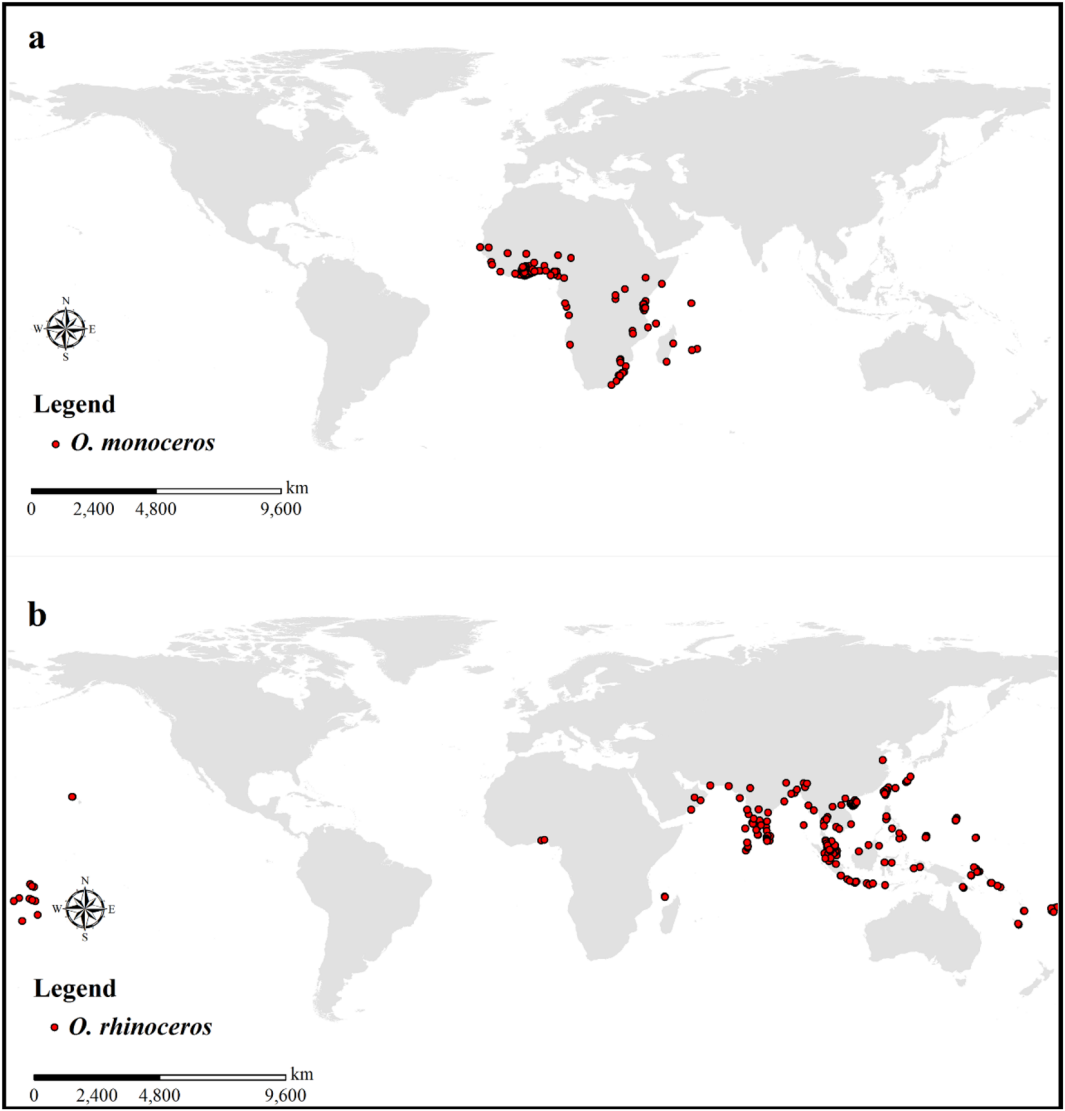
Occurrence records of *O. monoceros* and *O. rhinoceros.* ESRI ArcMap 10.2.2 (https://support.esri.com/en/Products/Desktop/arcgis-desktop/arcmap/10-2-2#downloads).

A 3-year nationwide survey to collect field data was conducted in palm plantations in Ghana for *O. monoceros*. The locations where *O. monoceros* was found were geolocated using a handheld GPS device. The presence of any of the developmental stages (i.e., eggs, larvae, pupae, and adults) and damaged symptoms were considered the existence of the pest. Since it is critical to have enough data points for accurate modelling, the field data were enriched by an extensive scientific literature search utilizing online databases such as Web of Science, Science Direct, Google, Google Scholar, PubMed, and MEDLINE (Supplemental information Table S1). The locations of occurrence *O*. *rhinoceros* were obtained from scientific literature by an extensive article search utilizing online databases such as Web of Science, Science Direct, Google, Google Scholar, PubMed, and MEDLINE (Supplemental information Table S1) by searching online using keywords, i.e., *Oryctes rhinoceros* and *O. rhinoceros*. East longitudes and north latitudes were transformed to positive values, while west longitudes and south latitudes were converted to negative values^27,33,42^. The latitudes and longitudes were proofread for accuracy using Google Earth. Duplicate records, fuzzy records, and neighboring records were all eliminated based on MaxEnt's requirements^43^. Overall, 322 and 304 occurrence records for *O. monoceros* and *O. rhinoceros,* respectively, were used for mapping the global geographical distribution of the two pests (Fig. 2).

### Environmental variables

Environmental variables affect the habitat and ecological niche of species^27^. These variables, together with the occurrence records, were used to model and map the spatial distribution of the two pests. The environmental variables consisted of climatic variables, as well as human, and geographical factors (Table 1).

**Table 1:** Nineteen (19) variables used for mapping the global distribution of *O. monoceros* and *O. rhinoceros* with the code and units.

Categories	Environmental variables	Code/unit	Data source
Climatic factors	Annual mean temperature	Bio1 (°C)	WorldClim version 2
	Mean diurnal range (mean of monthly (max temp–min temp))	Bio2 (°C)	
	Isothermality (Bio2/Bio7) (× 100)	Bio3 (°C)	
	Temperature seasonality (standard deviation × 100)	Bio4 (°C)	
	Max temperature of warmest month	Bio5 (°C)	
	Min temperature of coldest month	Bio6 (°C)	
	Temperature annual range (Bio5-Bio6)	Bio7 (°C)	
	Mean temperature of warmest quarter	Bio8 (°C)	
	Mean temperature of coldest quarter	Bio9 (°C)	
	Annual precipitation	Bio10(mm)	
	Precipitation of wettest month	Bio11 (mm)	
	Precipitation of driest month	Bio12 (mm)	
	Precipitation seasonality (coefficient of variation)	Bio13 (mm)	
	Precipitation of wettest quarter	Bio14 (mm)	
	Precipitation of driest quarter	Bio15 (mm)	
Geographical factors		Elevation (m)	Shuttle radar topography mission (SRTM)
		Vegetation	Global inventory modelling and mapping studies (GIMMS) group
Human factors		Land cover	International geosphere-biosphere programme (IGBP)
		Urban accessibility	European Commission Joint Research Center

The bioclimatic variables used for the mapping were obtained from the Global Climate Data website (Version 2.0, http://www.worldclim.org/), spanning 30 years from 1970 to 2000. The NASA Surface Meteorology and Solar Energy (https://eosweb.larc.nasa.gov/) global mean annual relative humidity datasets were transformed from a shape file to a raster layer as one of our input data, and 2.5 arc minutes with a spatial resolution of 5 km. As for the geographical factors, previous work showed that vegetation affected infestations of *O. rhinoceros* in young oil palms replanting in Malaysia^44^. It was also observed that some insects are limited to a small range of altitudes, whereas others are found in a large range of elevations^45^. Thus, we included vegetation and elevation as the geographical factors in the mapping of the two pests. In a series of four studies, researchers observed that cover crops reduced the abundance of the beetles^44^. Moreover, Normalized Difference Vegetation Index (NDVI) are commonly used to represent the development of plant canopy^21,46–49^. Therefore, NDVI dataset with a 5 × 5 km spatial resolution and a 15-day interval temporal resolution, obtained from Mapping Studies (GIMMS) group (http://glcf.umd.edu/), were used as a substitute of vegetation for this work. We computed the mean yearly NDVI using these datasets from 1982 to 2015 and used it as one of the input layers for machine learning models^21^. Land cover also affects the diversity and distribution of pests^50^. Because thermal accumulation drives development in many ectothermic species, elevated urban temperatures have the most pronounced consequences on ectotherms^51,52^. In this study, we used Pearson correlation to remove collinearity among predictor variables and those with correlation coefficients |r|≥ 0.7 were excluded from the final model (Table 2). As a result, we were able to select 10 environmental variables for the final simulation (Table 3).

**Table 2:**
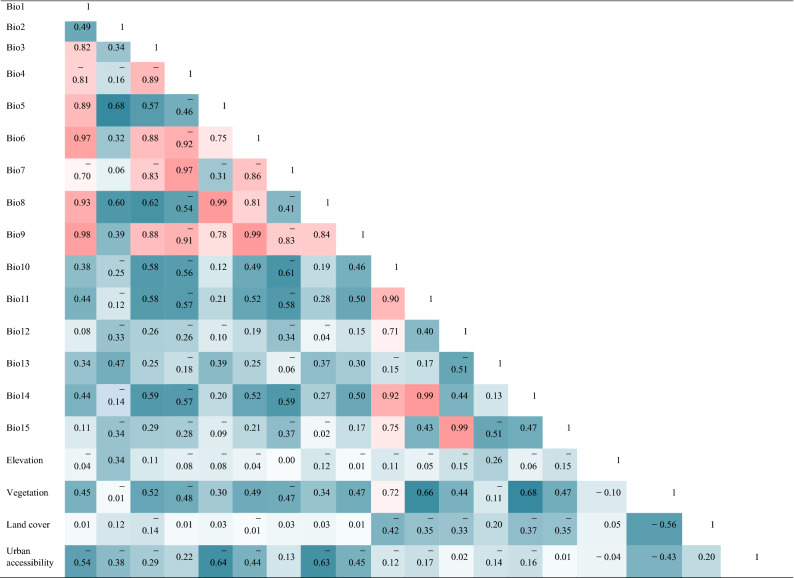
Correlation between environmental variables for the final model of *O. monoceros* and *O. rhinoceros*.

The depth-colored shading reflects the level of correlation between variables below 0.7 (blue) and more than 0.7 (including 0.7) (red).

**Table 3:** The environmental variables for modeling.

Categories	Environmental variables
Climatic factors	Bio2
	Bio7
	Bio8
	Bio11
	Bio12
	Bio13
Geographical factors	Elevation
	Vegetation
Human factors	Land cover
	Urban accessibility

### Modeling analysis

MaxEnt is a machine learning algorithm that employs the theory of maximum entropy^25–27^. This model relies on known occurrence records of an organism and corresponding environmental variables to analyze and predict the geographical distribution of the organism when the entropy reaches the highest point under limited conditions^27^. The entropy formula can be defined as:

1 $$H\left( {\hat{\pi }} \right) = { } - \mathop \sum \limits_{x \in X} \hat{\pi }\left( x \right)ln\hat{\pi }\left( x \right)$$

ln this natural logarithm, π as the unknown probability distribution over a finite set of pixels X within the study area is approximated by $\widehat{\pi }$. For each x, $\widehat{\pi }$ must be assigned a non-negative probability, when the integration of all the probabilities must equal one.

In this study, MaxEnt software version 3.4.1 was used to quantify the potential global distribution of *O*. *monoceros* and *O*. *rhinoceros*. The model has the advantage of being user-friendly for only requiring a small sample size to make accurate predictions^53,54^. In this study, 100 repetitions of the ten-fold cross-validation were performed for *O*. *monoceros* and *O*. *rhinocero*s in MaxEnt, respectively, and every model was run for 500 iterations to reduce model uncertainty. The area under the curve (AUC) and true skill statistic (TSS) were used to quantify the accuracy of the approach^55,56^. The AUC value lies from 0 to 1, with a value closer to 1 indicating higher model prediction accuracy, whereas an AUC value greater than 0.7 corresponds to better performance^55^. The TSS value ranges from − 1 to 1, and the closer the value is to 1, the better the prediction is, with ranges between 0.6 and 1 indicating a higher model accuracy^56^. The model estimates the contribution of the different environmental variables to the geographical distribution of the pests, and the mechanism by which the variables specifically affect the distribution is presented through the marginal response curve. To quantify the areas suitable for both *O. monoceros* and *O. rhinoceros*, we employed ArcGIS software’s inbuilt Tabulate Area tool (version 10.1)^57,58^.

### Ethical approval

This article does not contain any studies with human participants or animals performed by any of the authors.

## Results

### Prediction accuracy of the MaxEnt model

**Figure 3:**
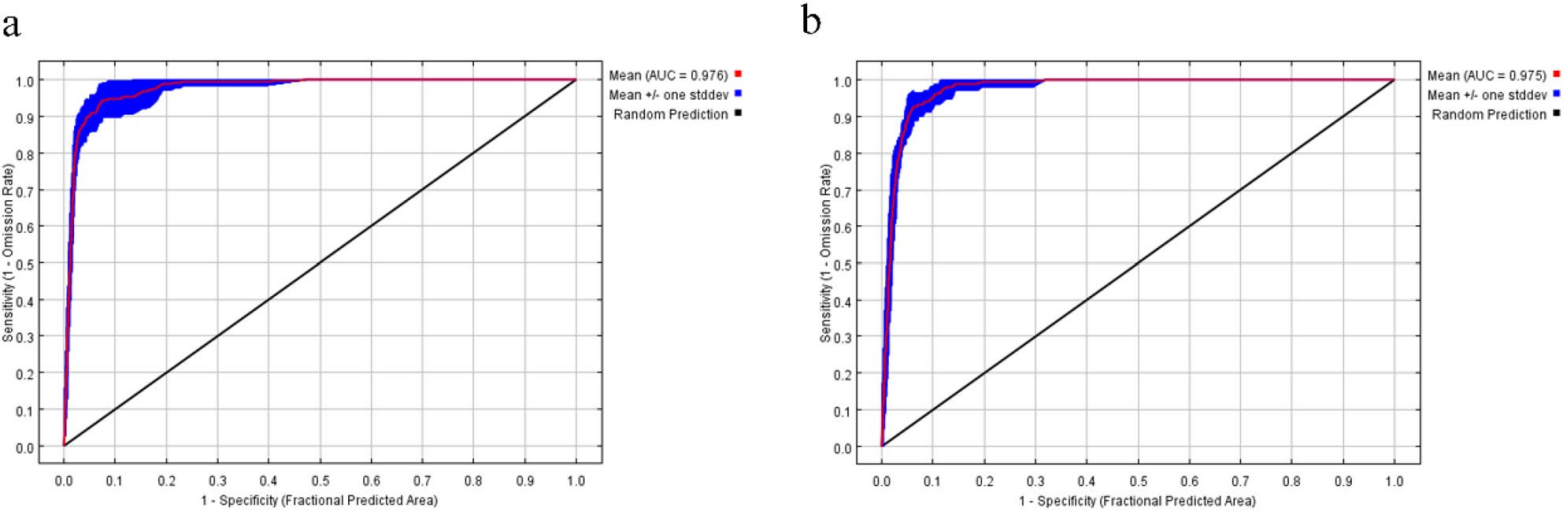
Receiver operating characteristics (ROC) curves and area under the curve (AUC) values of the MaxEnt models: (**a**) *O. monoceros*; (**b**) *O. rhinoceros*.

The projected spatial distribution results and the actual distribution of *O. monoceros* and *O. rhinoceros* have a high degree of overlap, indicating that the results can be applied to the appropriate regionalization of these species (Fig. 2a and b). The MaxEnt model performance was determined using the distribution points of *O. monoceros* and *O. rhinoceros*, and 10 environmental variables. The test data of AUC for *O. monoceros* and *O. rhinoceros* were 0.976 and 0.975, respectively (Fig. 3a and b). The TSS value for *O. monoceros* was 0.90, whereas 0.88 was obtained for *O. rhinoceros*.

### Global potential distribution of *O*. *monoceros* and* O*. *rhinoceros*

**Figure 4:**
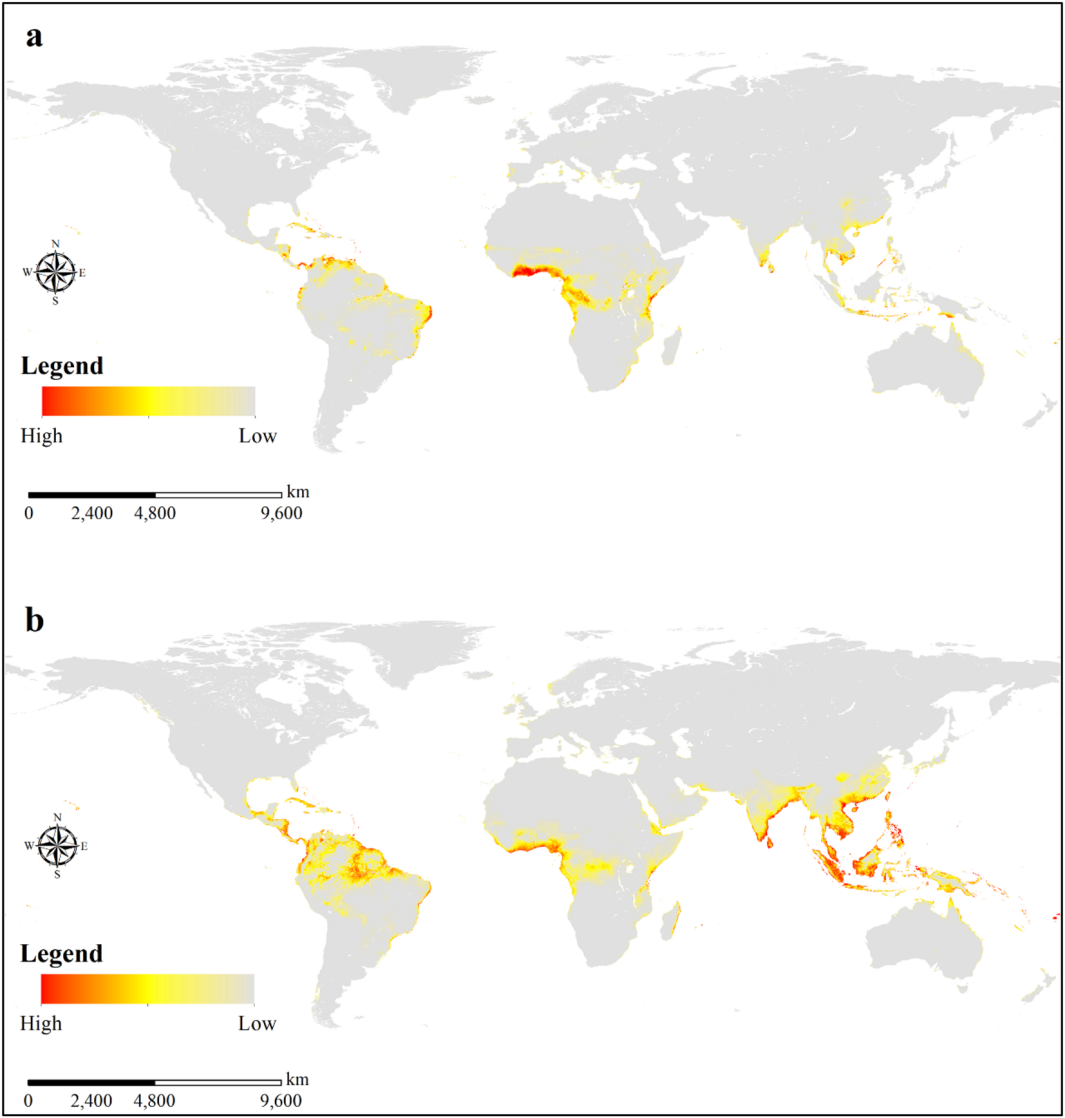
Global potential distribution maps generated by MaxEnt for: (**a**) *O. monoceros*; (**b**) *O. rhinoceros.* MaxEnt 3.4.1 (https://biodiversityinformatics.amnh.org/open_source/maxent/) and ESRI ArcMap 10.2.2 (https://support.esri.com/en/Products/Desktop/arcgis-desktop/arcmap/10-2-2#downloads).

The simulated suitable areas for *O. monoceros* and *O. rhinoceros* covered the present-day known occurrence records of the pests (Fig. 4a and b). The model predicts overlaps of highly suitable areas for *O. monoceros* and *O. rhinoceros*, especially in parts of the north and east coasts of South America; west, south, and east coasts of Africa; south and east coasts of Asia; and a few suitable areas scattered in the coastal regions of Northern Oceania. The mapping shows that parts of Ghana, Nigeria, Tanzania, Mozambique and Côte d'Ivoire in Africa; Indonesia, India, Malaysia, Thailand, and the Philippines in Asia; Brazil, Venezuela and Colombia in the Americas; and Papua New Guinea in Oceania, that produce large quantities of palms are also suitable for the two pests. However, the results show that suitable global areas for *O. rhinoceros* (1279.00 × 10^4^ km^2^) are greater than that of *O. monoceros* (610.72 × 10^4^ km^2^) (Table 4).

**Table 4:** The suitable area (ten thousand km^2^) of *O. monoceros* and *O. rhinoceros* by continent.

Continent	Suitable area (ten thousand km^2^)
***O. monoceros***	
Asia	137.09
South America	150.24
Africa	269.80
North America	32.98
Australia	9.94
Oceania	2.84
Europe	7.83
Antarctica	0.00
Total	610.72
***O. rhinoceros***	
Africa	223.58
Antarctica	0.00
Asia	610.63
Australia	15.40
Europe	6.59
North America	70.13
Oceania	13.36
South America	339.32
Total	1279.00

**Figure 5:**
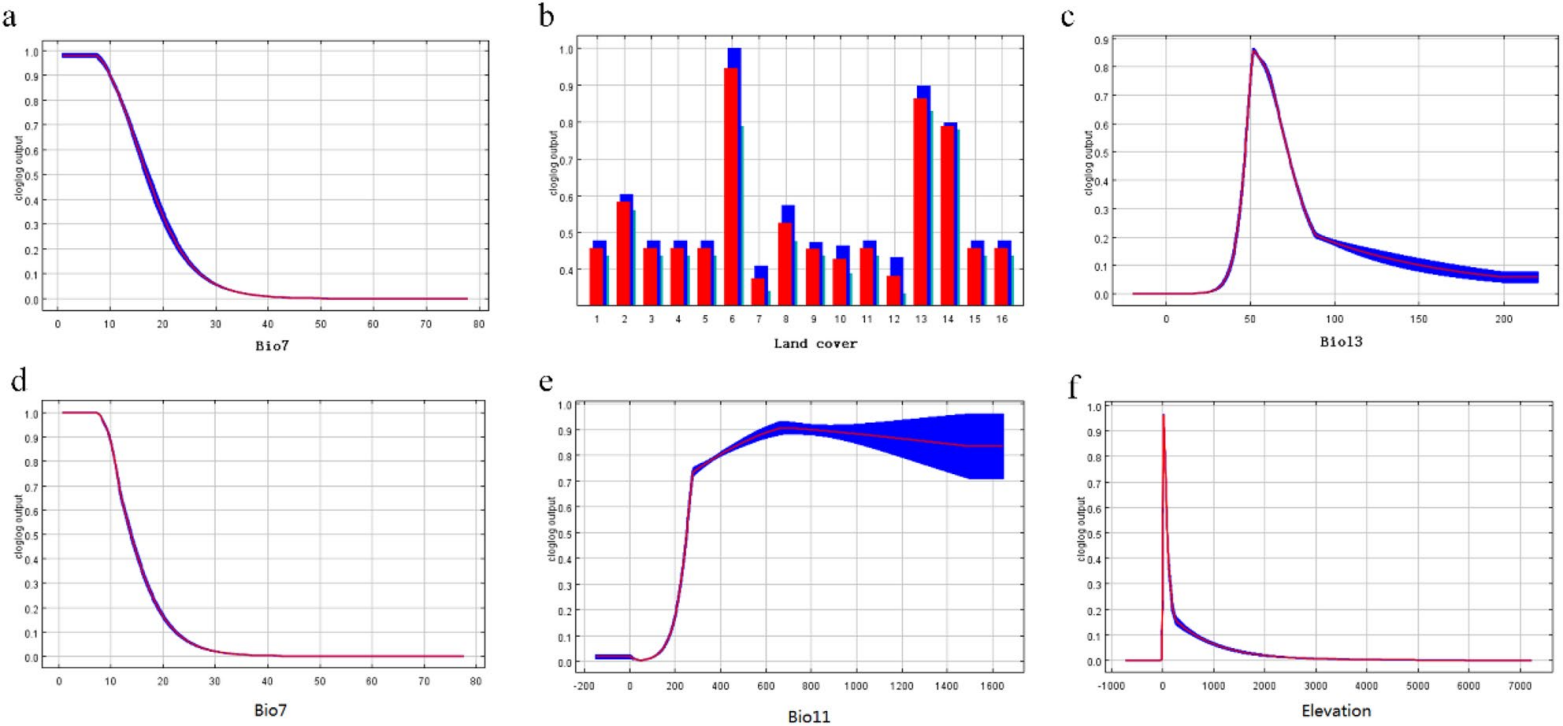
Response curves of the most significant environmental variables in mapping suitable areas of *O. monoceros* (**a**–**c**) and *O. rhinoceros* (**d**–**f**). Note: In part b, 1–16 means evergreen needleleaf forests, evergreen broadleaf forests, deciduous needleleaf forests, deciduous broadleaf forests, mixed forests, closed shrublands, open shrublands, woody savannas, savannas, grasslands, permanent wetlands, croplands, urban and built-up lands, cropland/natural vegetation mo-saics, permanent snow and ice, and barren, respectively.

The response curves of the three most important environmental variables with regard to their suitability for the prediction of *O. monoceros* and *O. rhinoceros* global distribution are shown in Fig. 5. The response curve for temperature annual range shows that there is less chance of *O. monoceros* colonisation when temperature annual range increases (Fig. 5a). The predictive probability of the *O. monoceros* to the land cover variable demonstrates that the closed shrublands is the most critical class determining the geographic suitability for *O. monoceros* occurrence (Fig. 5b). Moreover, the predictive probability of the presence of *O. monoceros* colonisation was high when precipitation seasonality ranged between 40 and 80 mm (Fig. 5c). With *O. rhinoceros*, the predictive probability of its geographic suitability decreased when temperature annual range increases (Fig. 5d), while probability of colonisation increases with an increase of precipitation in wettest month (Fig. 5e). The response curve obtained for elevation showed that the predictive probability of *O. rhinoceros* colonisation was high at elevations ranging between 0 and 2000 m (Fig. 5f).

### Contribution of environmental variables

Our results showed that the temperature annual range (Bio7, 47.2%) contributed most to the *O. monoceros* model, followed by land cover (31.2%), precipitation seasonality (Bio13, 6.1%), and precipitation of wettest month (Bio11, 5.5%) (Table 5). The cumulative contribution of the four variables was 90%. For *O. rhinoceros*, temperature annual range (Bio7, 33.6%) contributed most to the model, followed by precipitation of wettest month (Bio11, 28.6%), elevation (14.2%), and land cover (8.8%). The four factors contributed 85.2% in total to the *O. rhinoceros* model (Table 5).

**Table 5:** Environmental variables considered in *Oryctes monoceros* and *O. rhinoceros* niche models and mean percentage contribution of environmental variables in the models; values were averaged over ten repeated runs.

Environmental variables	Relative contribution (%)
***O. monoceros***	
Bio7	47.2
Land cover	31.2
Bio13	6.1
Bio11	5.5
Vegetation	3.8
Bio12	2.8
Bio2	2.4
Elevation	0.5
Bio8	0.4
Urban accessibility	0.1
***O. rhinoceros***	
Bio7	33.6
Bio11	28.6
Elevation	14.2
Land cover	8.8
Bio8	4.8
Bio2	3.8
Urban accessibility	2.8
Vegetation	1.6
Bio12	1
Bio13	0.8

## Discussion

Ecological niche modeling has provided insight into identifying areas suitable for species and estimating the potential impact of environmental variables on their geographical distribution^55^. It has been quite helpful for mapping the niche shifts of plant diseases^59,60^, pests^61^, and plants^62,63^. In the present study, we conducted ecological niche modelling by combining MaxEnt with elevation of *O. monoceros* and *O. rhinoceros*, the two most harmful pests to worldwide palms.

We obtained the global potential distribution maps of *O. monoceros* and *O. rhinoceros* from the MaxEnt modeling. The prediction accuracy for the two pests based on AUC and TSS was good^61,64^, suggesting that the quantification of the geographical distribution was reliable and could provide critical guidance for policy formulation and mitigation measures, especially in regions where the pests have not emerged yet. We also generated the predicted global distribution map of the two pests using aeronautical reconnaissance coverage geographical information system. The tool has been applied in mapping the geographical distribution of pests, such as *Spodoptera frugiperda* (J.E.Smith) (Lepidoptera: Noctuidae) in central Asia^65^, *Bactrocera dorsalis* Hendel (Diptera: Tephrididae) in China^66^, and *Episimus utilis* Zimmerman (Lepidoptera: Tortricidae) in Brazil^67^.

Climate change, playing a significant role in the dispersal and outbreaks of agricultural pests, has been associated with the rise in temperature^68^. Recent climate models predict a 1 °C increase in global mean annual temperatures by 2025 and a potential 3 °C rise by the end of the century^69^. As a result, the anthropogenically induced climatic change caused by increased quantities of the earth’s atmospheric greenhouse gases is projected to impact agricultural pests considerably^70^. Our study analyzed the important environmental variables that influence the distribution of *O. monoceros* and *O. rhinoceros* based on the MaxEnt model. Our models’ predictions identified temperature annual range, followed by land cover, and then precipitation seasonality as the most important environmental variables that determined the distribution of *O. monoceros*. In contrast, Aidoo et al.^58^ reported that annual temperature variation, followed by seasonality of temperature, then isothermality, were the main environmental variables determining the distribution of *O. monoceros* based on Boosted regression tree (BRT) model.

According to the response curve, closed shrublands were the most important land cover variable affecting the distribution of *O. monoceros*. The presence of dead woods and dead palm trunks serve as a breeding site for the pest, whereas cover crops serve as a barrier preventing the beetles from identifying breeding sites^10^. For *O. rhinoceros*, temperature annual range contributed most to the prediction, followed by annual precipitation, suggesting that the temperature condition is more important than rainfall condition in defining the distribution of the pest. In contrast, minimum temperature of coldest month, followed by precipitation of wettest month were reported by Hao et al.^57^ as the main drivers of the global distribution of *O. rhinoceros* based on BRT model. Xu et al.^71^ reported that the precipitation of the wettest month was the most important driver of the potential distribution of *O. rhinoceros*. The variation in the modeling results could be associated with the modeling technique, environmental variables and the number of *O. rhinoceros* distribution points. Nevertheless, previous studies have shown that temperature affects the biology of *O. rhinoceros*, and the preferred temperature for the development and survival of *O. rhinoceros* ranges from 27 to 29 °C and relative humidity ranging from 85 to 95%^72,73^.

Temperature is one of the most important abiotic factors affecting the growth, development, reproduction, and survival of insects^74^. In this study, we found that temperature annual range was the most critical environmental variable for both beetles. The impact of temperature on insect growth varies with the species, but it is quite certain that lower temperatures usually result in a slower rate of development^75^. More than that, temperature affects a variety of biological properties of insects, including the sex ratio^76^, adult lifespan, survival, fecundity, and fertility^77^, leading to a significant impact on insect colonization, distribution, abundance, behavior, life history, and fitness^78,79^.

The risk map of *O. monoceros* shows a potential expansion to suitable areas outside its current known distributed areas, notably in Latin America and Asia. Similarly, the simulation of potential areas suitable *O. rhinoceros* also covers areas outside the current distribution of the pest. These areas include parts of West and East Africa, Oceania, and Latin America. The globalization of the international horticulture plant trade has increased the risk of inadvertent spread of leaf beetles from their original geographic locations to uninfected areas^80^. The unintentional transport of plant materials on agricultural equipment such as farm machinery and tools may be the most likely mode of spread to areas that the pest has not yet arrived. The record of *O. monoceros* in Yemen^17^ suggests that the pest can invade new regions, including Asia and other regions as same as we predicted in our study. An earlier study associated with the spread of *O. rhinoceros* from South and Southeast Asia to Guam to the transport of commercial soil products^19^. It is therefore imperative for stakeholders and plant regulatory services and NGOs to take an interest in safeguarding the oil palm, coconut, and date palm industry to stay alert for the pests as well as devising countermeasures for the control and prevention of *O. monoceros* and *O. rhinoceros*, especially in uninvaded areas.

Our findings showed that the total areas suitable for *O. monoceros* was 610.72 × 10^4^ km^2^. Of this area, the percentage of suitable area in the native range was slightly lower than that of the predicted habitat suitability. Specifically, our simulation results show that about 55.8% of the predicted suitable areas were found outside the native range of *O. monoceros*. In general, the global suitable areas for *O. monoceros*, as simulated in this study, was slightly lower than that of Aidoo et al.^58^. The observed variation may be that the present study included human, climatic and geographic factors in the present simulation, while Aidoo et al.^58^ considered only climatic and geographic factors. Notwithstanding, there also some similarities in the areas predicted to be suitable for *O. monoceros* in both studies. The simulation for *O. rhinoceros* using the BRT model show that there are suitability in the major palm producing countries^57^ which is consistent with MaxEnt modeling, as suitable areas are found in Indonesia, Malaysia, Tanzania, India, Philippines and Brazil. In the present study, suitable areas for *O. rhinoceros* was found to be 1279.00 × 10^4^ km^2^ with about 52.3% habitat suitability outside its native range. These predicted areas are less than that of Hao et al.^57^, and the variations could be associated with the input data, such as land cover, urban accessibility and vegetation. It could also be due to the modeling approach used for the different studies. Previous studies showed that different modeling methods affected ecological niche predictions of species differently^81,82^.

In this study, urban accessibility was the least (0.1%) important variable influencing the distribution of *O. monoceros*, while the same variable ranked seventh (2.8%) among the most important variables determining the distribution of *O. rhinoceros*. The results showed that the relative contribution of elevation to *O. monoceros* and *O. rhinoceros* models were 0.5 and 14.2%, respectively. While the relative contribution of vegetation to *O. moncoeros* model was 3.8, 1.6% was obtained for *O. rhinoceros*. Moreover, the relative contribution of urban accessibility for *O. monoceros* differed from that of *O. rhinoceros*, with the latter contributing to 2.8% of its model. This, however, suggests that the two species responded differently to these human and geographical factors and had a significant influence on the extent of the geographical distribution of the two species, as illustrated in the risk maps. The effects of land cover, urban accessibility, elevation, and NDVI could also influence host distribution through their effects on host food and habitats. For example, species' habitat requirements are likely to be influenced by the presence of nearby bushes^83^. Moreover, vegetation on the ground, the effect of cover crops like natural, legume, or grass, and the presence of bare ground all have an impact on the number of beetles in their breeding sites^84^.

## Conclusions

For the first time, we have combined MaxEnt with elevation, vegetation, urban accessibility, land cover, and bioclimatic variables to determine the potential geographical distribution of *O. monoceros* and *O. rhinoceros*. Our study has created risk maps for the two major pests of palms to facilitate decision-making and the timely launching of preventive measures. The risk maps identify suitable areas outside the currently distributed regions of the pests. In this study, we found that thermal conditions were the most important factors governing the spread of pests.

## Supplementary Information

Supplementary Information.

## Data Availability

All data generated or analysed during this study are included in this published article and the supplementary information files.

## Code Availability

Please contact the corresponding author for code of decent request.
